# Racial inequities in second-line treatment and overall survival among patients with metastatic breast cancer

**DOI:** 10.1007/s10549-022-06701-5

**Published:** 2022-08-26

**Authors:** Kristen D. Whitaker, Xiaoliang Wang, Mustafa Ascha, Timothy N. Showalter, Heather G. Lewin, Gregory S. Calip, Lori J. Goldstein

**Affiliations:** 1 Fox Chase Cancer Center, 333 Cottman Avenue, Philadelphia, PA 19111 USA; 2 Flatiron Health, Inc, 233 Spring Street 5th Floor, New York, NY 10013 USA; 3 University of Virginia School of Medicine, Charlottesville, VA USA; 4 University of Illinois at Chicago College of Pharmacy, Chicago, IL USA

**Keywords:** Metastatic breast cancer, Endocrine therapy, CDK 4/6, Inequities

## Abstract

**Background:**

Black women in the USA have a higher incidence and mortality of metastatic breast cancer (mBC) than White women, while Hispanic women have lower rates. Previous studies have focused on first-line (1L) treatment, but little is known about racial differences in treatment beyond 1L and their impact on outcomes.

**Methods:**

This analysis utilized data from an electronic health record derived de-identified database and included patients with HR+HER2- mBC initiating 2L treatment (including CDK4/6-inhibitor [CDKi]-based, endocrine monotherapy, everolimus combination therapy, and chemotherapy and other systemic therapies) between 2/3/2015 and 7/31/2021. Real-world overall survival (rwOS) was defined as time from 2L initiation to death. Multinomial logistic regression assessed the likelihood of 2L treatment between race/ethnicity groups. Median rwOS was estimated using the Kaplan–Meier method and adjusted hazard ratios were estimated using multivariable Cox proportional hazards models.

**Results:**

Among all patients who received 2L, non-Hispanic Black (NHB) and Hispanic/Latino patients were less likely to receive 2L CDKi compared to non-Hispanic White (NHW) patients (36%, 39% vs 42%, respectively). Median rwOS was 20.4, 37.6, and 25.3 months, in NHB, Hispanic/Latino and NHW patients, respectively. The rwOS remained poorer among NHB patients after adjustment (HR = 1.16; *p* = 0.009). In stratified analysis, adjusted rwOS was similar between NHB and NHW patients among those who received 1L CDKi.

**Conclusions:**

These findings suggest that among patients with HR+HER2- mBC, NHB patients had worse survival beyond front-line setting, mainly among the subset of women who did not receive CDKi at 1L. This inequities in rwOS between race/ethnicity groups was not observed among patients who received 1L CDKi.

**Supplementary Information:**

The online version contains supplementary material available at 10.1007/s10549-022-06701-5.

## Introduction

In the USA, African American or Black (AA) women not only have a higher incidence of metastatic breast cancer (mBC) but also increased breast cancer mortality compared to White women [1, 2]. Although improved breast cancer screening and treatments have resulted in an overall decrease in the breast cancer mortality, the divergence in mortality trends between Black and White women remains large in the USA [3]. In contrast, both incidence and survival rates of mBC were lower among Hispanic/Latina women compared to White women [1]. The drivers of racial inequities in mortality are multifactorial and may include more aggressive tumor characteristics, unequal access to care, and poorer socioeconomic status, among AA women with BC, although previous cohort study using the Surveillance, Epidemiology, and End Results (SEER) database found that the disproportionate burden of death in AA women persisted in subgroups with higher socioeconomic status and less aggressive tumor types [2].

The systemic treatment of women with hormone receptor (HR) positive, HER2 negative (HR+HER2−) mBC has historically involved primary endocrine therapy (ET) with selective use of chemotherapy [4]. In the past, ET-based regimens were the preferred treatment option in the 1L, 2L, and subsequent-line settings [5]. However, resistance to ET usually leads to disease progression and ultimately deaths from breast cancer [6]. Recent evidence has established the addition of cyclin-dependent kinase 4/6 inhibitor (CDKi) to ET as a preferred approach for 1L and subsequent treatments [7, 8]. Previous studies have shown patterns of care and possible health inequities in treatment delay [9], the use of CDKi as first-line (1L) treatments [2, 10, 11], and survival [12]. However, it is unknown whether the inequities persist after treatment initiation and beyond first-line treatment, and whether it may further result in inequities in patient outcomes. Meta-analysis of randomized clinical trials suggests that compared to ET alone, combination of a CDKi and ET improved overall survival (OS) in both 1L and 2L treatment subgroups [13]. However, trial subgroup analysis stratified by Black, Hispanic/Latino vs White race groups is not available in these trials [14]. Therefore, real-world evidence among patients with mBC treated in routine practice may provide important insights on this topic.

This study aims to investigate breast cancer treatment inequities across the care continuum for women with HR+HER2− mBC by examining differences in 2L treatment options and real world overall survival (rwOS) across racial or ethnic groups. We evaluate the use of CDKi, ET and chemotherapy in 2L setting, and rwOS from 2L initiation between race/ethnicity groups.

## Methods

### Data source and population selection

This study used the nationwide Flatiron Health electronic health record (EHR)-derived de-identified database. The Flatiron Health database is a longitudinal database, comprising de-identified data originating from approximately 280 US cancer clinics (800 sites of care) [15, 16]. Included patients were adult patients with confirmed diagnosis of mBC on or after 1/1/2011, who had at least two EHR-documented visits on or after 1/1/2011 and had received at least two lines of therapy. Patients also must have had HR + /HER2− disease as identified by any positive HR and only negative HER2 status test results measured on or before 1L initiation. Patients were excluded if they were male, had unknown dates or results of biomarker tests only, had a > 90 day gap between metastatic diagnosis and first documented visit (in order to minimize risk of missing treatment data), or if they received clinical study drugs for 1L or 2L treatment. To ensure assessment of CDKi exposure within the era when such drugs were widely available, only patients who initiated 2L treatment for mBC between 2/3/2015 (first FDA approval of a CDKi) and 7/31/2021 (at least six months before the end of follow-up at Jan 31, 2022) were included in the analysis.

Institutional Review Board approval of the study protocol was obtained prior to study conduct, and included a waiver of informed consent.

### Variables and endpoints

The primary exposure variable was documented race/ethnicity, categorized as Non-Hispanic White (NHW; reference group), Non-Hispanic Black (NHB), Hispanic or Latino, and Other/Unknown. Patient-level demographic and clinical characteristics were ascertained using structured and unstructured data, curated via technology-enabled abstraction. Key covariates include: age at metastatic diagnosis (years), stage at initial diagnosis (stage I–III, stage IV and unknown), practice type (community vs academic), 1L treatment group (CDKi, endocrine therapy, or chemotherapy and others)^5^, duration of 1L treatment (days), progression during 1L (yes/no), progression within the first 6 months of 1L start (yes/no), ECOG performance status at the start of 2L treatment, insurance status, and number and sites of metastasis on or before 2L start. ECOG performance status was categorized as 0, 1, ≥ 2, and unknown, based on the ECOG value measured within 30 days prior to and 7 days after 2L initiation. Insurance status was categorized as commercial health plan payer (reference group), Medicaid, Medicare, self pay/undocumented, and other payer, based on the last insurance record before 2L initiation. Site of metastasis was categorized as visceral, bone only and others. Number of metastases was categorized as 1, 2, and ≥ 3. In addition, distribution of area-level socioeconomic status (SES) index (quintile) were also summarized by race/ethnicity group. The SES index was constructed using a factor analysis from seven American Community Survey (ACS, 2015–2019) characteristics of census block group-level social determinants of health following the approach of Yost [17, 18]. Missing values of the variables that were used for constructing SES index were imputed using multiple imputation by chained equations [19]. Additional categories of unknown were used for variables with missing values, e.g. ECOG PS, insurance, SES index.

The primary outcomes were 2L treatment groups and real-world overall survival (rwOS). Treatment groups at 2L were categorized as: (1) any CDKi (monotherapy or combination therapies); (2) endocrine therapy alone; (3) everolimus combination therapy; and, (4) chemotherapy and others (reference group). The rwOS was defined as time from start of 2L treatment to date of death, or censoring at last confirmed activity [20]. Vital status (alive/death) and date of death were determined using de‐identified patient‐level structured and unstructured data from the EHR, curated via technology‐enabled abstraction, obituary data, and the public Social Security Death Index [20].

### Statistical analyses

Descriptive statistics were used to compare demographic and clinical characteristics at 2L between race/ethnicity groups. Rates of receiving 2L treatment groups were estimated by race/ethnicity, and further stratified by 1L treatment (CDKi, vs chemotherapy/ET). Multinomial logistic regression was used to assess the likelihood of 2L treatment groups between NHB, Hispanic/Latino, Other/Unknown, and NHW (reference) patients, adjusted for demographics and clinical factors. For survival analysis, median rwOS was estimated using the Kaplan–Meier method and log-rank test. Adjusted hazard ratios (aHR) between race groups were estimated using multivariable Cox proportional hazards models, additionally adjusted for 2L treatment. Stratified analysis was conducted by 2L treatment groups. Sensitivity analyses omitting variables with high levels of missingness (ECOG status and insurance) and excluding patients with missing values in these variables were performed for both primary analyses. In exploratory analysis, we further stratified the survival analysis by 1L treatment (CDKi vs chemotherapy/ET) and 2L treatment groups.

All analyses were conducted using R version 3.6.1.

## Results

### Baseline characteristics

A total of 5849 patients (3792 NHW; 611 NHB, 467 Hispanic/Latino, 979 Other/Unknown) with HR+HER2− mBC were included in this study. Compared to NHW patients, NHB and Hispanic/Latino patients were more likely to be younger at metastatic diagnosis (median age: 60 years in NHB, 61 years in Hispanic/Latino, vs 65 years in NHW), have Medicaid (15% in NHB and Hispanic/Latino, vs 6% in NHW), live in the least affluent SES area (30% in NHB, 33% in Hispanic/Latino, vs 9% in NHW). treated at community practices (91% vs 87% in White), and have shorter duration of 1L therapy (median 119 days in NHB, 106 in Hispanic/Latino, vs 160 in NHW). NHB patients were also more likely to have de novo diseases (29% vs 26% in NHW), have visceral metastases (53% vs 46% in NHW), and were more likely to progress within 6 months of treatment initiation during 1L therapy (28% vs 22% in NHW; Table 1). Lower rates of 1L CDKi use were observed among NHB (29%), Hispanic/Latino (27%), compared to NHW (31%) patients. Endocrine therapy (alone and in combination with targeted therapy) was less frequently administered at 1L, and chemotherapy and other systemic therapies more often, for patients in the race/ethnicity groups other than NHW.

**Table 1:** Baseline characteristics among patients with HR+HER2- metastatic breast cancer by race/ethnicity

	Non-Hispanic White	Non-Hispanic Black	Hispanic/ Latino	Other/ unknown	*p*-value
	*N* = 3792	*N* =611	*N* = 467	*N* = 979	
Age at metastatic diagnosis [years, median (IQR)]	65.0 [56.0;74.0]	60.0 [51.0;70.0]	61.0 [51.0;71.0]	63.0 [54.0;71.0]	< 0.001
Practice type:					< 0.001
Academic (with or without community)	476 (12.6%)	55 (9.0%)	40 (8.6%)	45 (4.6%)	
Community	3316 (87.4%)	556 (91.0%)	427 (91.4%)	934 (95.4%)	
Stage at initial diagnosis					< 0.001
I—III	2524 (66.6%)	383 (62.7%)	301 (64.5%)	562 (57.4%)	
IV	986 (26.0%)	177 (29.0%)	112 (24.0%)	319 (32.6%)	
Not documented	282 (7.4%)	51 (8.3%)	54 (11.6%)	98 (10.0%)	
ECOG Performance Status					< 0.001
0	1219 (32.1%)	206 (33.7%)	169 (36.2%)	280 (28.6%)	
1	129 (34.0%)	220 (36.0%)	136 (29.1%)	297 (30.3%)	
≥ 2	524 (13.8%)	75 (12.3%)	40 (8.6%)	127 (13.0%)	
Missing	760 (20.0%)	110 (18.0%)	122 (26.1%)	275 (28.1%)	
Number of metastasis sites					0.155
1	1636 (43.1%)	259 (42.4%)	234 (50.1%)	420 (42.9%)	
2	1124 (29.6%)	179 (29.3%)	125 (26.8%)	292 (29.8%)	
≥ 3	1032 (27.2%)	173 (28.3%)	108 (23.1%)	267 (27.3%)	
Site of metastasis					0.008
Visceral	1731 (45.6%)	324 (53.0%)	205 (43.9%)	480 (49.0%)	
Bone only	1128 (29.7%)	143 (23.4%)	142 (30.4%)	274 (28.0%)	
Other	933 (24.6%)	144 (23.6%)	120 (25.7%)	225 (23.0%)	
Insurance Type					< 0.001
Commercial Health Plan	1638 (43.2%)	254 (41.6%)	174 (37.3%)	420 (42.9%)	
Medicaid	214 (5.6%)	93 (15.2%)	71 (15.2%)	86 (8.8%)	
Medicare	1218 (32.1%)	137 (22.4%)	69 (14.8%)	250 (25.2%)	
Other Payer	206 (5.4%)	38 (6.2%)	57 (12.2%)	58 (5.9%)	
Self-pay/Undocumented	516 (13.6%)	89 (14.6%)	96 (20.6%)	165 (16.9%)	
SES index (quintile)					< 0.001
Q1 (most affluent)	680 (17.9%)	41 (6.7%)	35 (7.5%)	212 (21.7%)	
Q2	694 (18.3%)	83 (13.6%)	46 (9.9%)	203 (20.7%)	
Q3	671 (17.7%)	84 (13.7%)	52 (11.1%)	161 (16.4%)	
Q4	537 (14.2%)	120 (19.6%)	65 (13.9%)	125 (12.8%)	
Q5 (least affluent)	354 (9.3%)	182 (29.8%)	155 (33.2%)	113 (11.5%)	
Unknown	856 (22.6%)	101 (16.5%)	114 (24.4%)	165 (16.9%)	
First-line treatment					< 0.001
CDKi	1191 (31.4%)	177 (29.0%)	127 (27.2%)	335 (34.2%)	
Endocrine therapy	1845 (48.7%)	267 (43.7%)	202 (43.3%)	433 (44.2%)	
Chemotherapy or others	756 (19.9%)	167 (27.3%)	138 (29.6%)	211 (21.6%)	
Duration of 1L (days; medium [IQR])	160.0 [60.0;461.2]	119.0 [49.0;339.0]	106.0 [56.0;293.0]	132.0 [59.5;346.5]	< 0.001
Real-world progression during 1L					0.008
Yes	2123 (56.0%)	335 (54.8%)	223 (47.8%)	528 (53.9%)	
No	1669 (44.0%)	276 (45.2%)	244 (52.2%)	451 (46.1%)	
Real-world progression within 6 months of 1L during 1L					0.010
Yes	844 (22.3%)	173 (28.3%)	111 (23.8%)	237 (24.2%)	
No	2948 (77.7%)	438 (71.7%)	356 (76.2%)	742 (75.8%)	
Follow-up status					< 0.001
Dead	2305 (60.8%)	381 (62.4%)	205 (43.9%)	545 (55.7%)	
Alive	1487 (39.2%)	230 (37.6%)	262 (56.1%)	434 (44.3%)	

*CDKi* cyclin-dependent kinase 4/6 inhibitor; *ECOG* Eastern Cooperative Oncology Group; *IQR* interquartile range

### 2L treatment characteristics

**Fig. 1:**
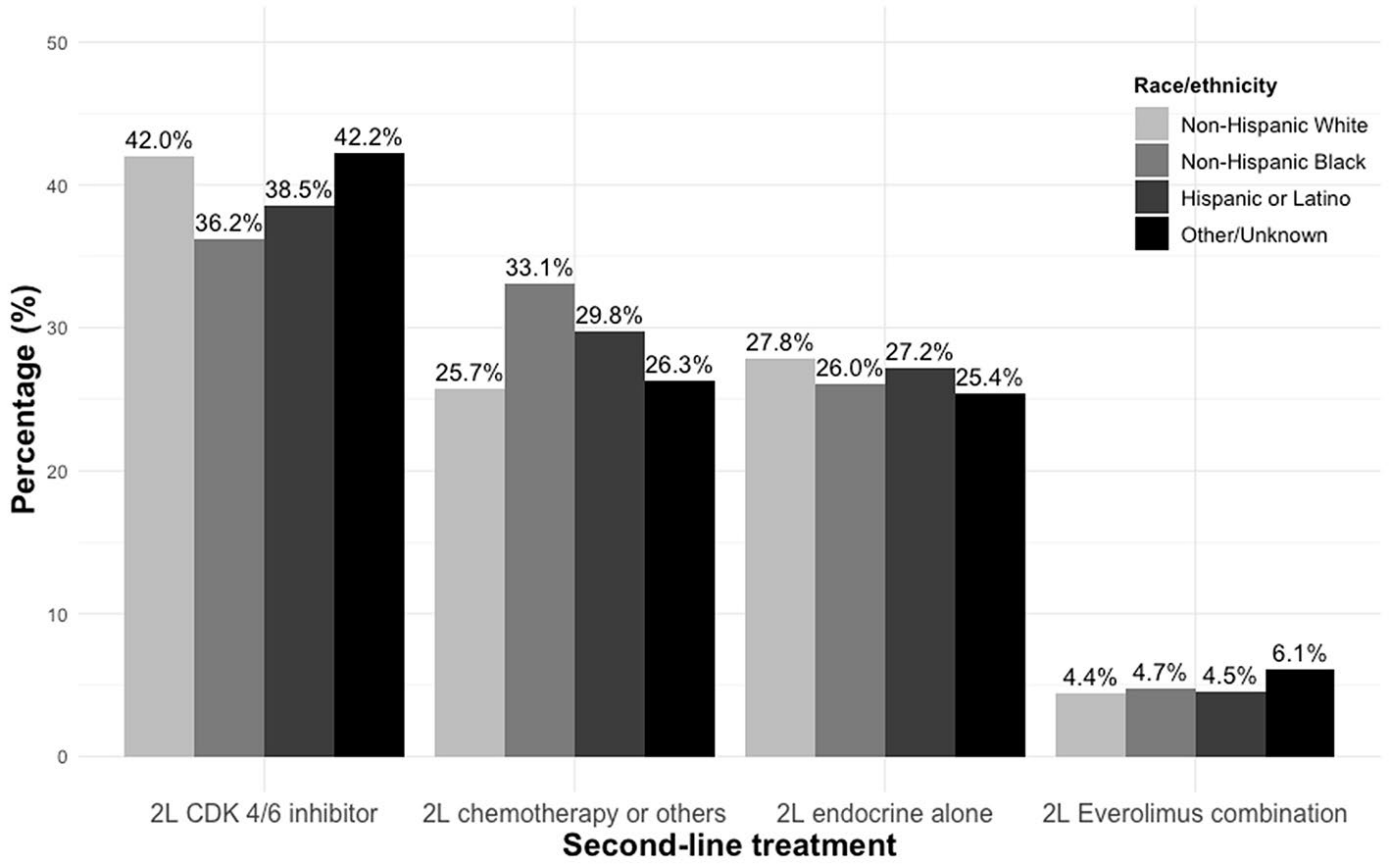
Histogram of 2L treatment groups by race/ethnicity

Among all patients who initiated 2L treatment, CDKi were administered as 2L treatment less often for NHB women (36%) and Hispanic/Latina women (39%) than for White women (42%), while chemotherapy was administered more often among NHB (33%) and Hispanic/Latina (30%) women than White women (26%; Fig. 1). Similar rates of endocrine monotherapy and everolimus combination therapy were administered as 2L treatment across race/ethnicity groups. In stratified analysis by use of 1L CDKi, among patients who did not receive CDKi at 1L, NHB and Hispanic/Latino patients were less likely, than NHW patients, to receive CDKi at 2L (NHB: 34%, Hispanic/Latino: 34% vs NHW: 43%). However, among patients who received CDKi at 1L, the rates of receiving CDKi at 2L were higher among Hispanic/Latino patients (50%), and similar among NHB (41%) and NHW (41%) patients (Supplemental fig. 1).

In univariate analysis, NHB and Hispanic/Latino patients had 33% and 21% lower odds of receiving 2L CDKi (vs. chemotherapy and others), compared to NHW patients (crude OR [cOR]: NHB: 0.67;95% CI: 0.54–0.82; Hispanic/Latino: 0.79; 95% CI: 0.63–1.00), respectively. After adjusting for demographic and clinical characteristics, the odds of receiving 2L CDKi (vs. chemotherapy and others) among NHB and Hispanic/Latino patients were 20%, and 10% lower than those among NHW patients [adjusted OR (aOR): NHB: 0.80; 95% CI: 0.63–1.02; Hispanic/Latino: 0.90; 95% CI: 0.68–1.19), respectively, but this difference was no longer statistically significant (Table 2). The odds of receiving different 2L treatment classes were similar between patients with other or unknown race/ethnicity, compared to NHW patients. Sensitivity analyses had similar results (Supplemental Table 1).

**Table 2:** Associations between race and likelihood of receiving different 2L treatment

Race	N	Unadjusted		Adjusted^a^	
		cOR (95% CI)	*p*-value	aOR (95% CI)	*p*-value
2L chemotherapy or other (reference group)					
Non-Hispanic White	974	–	–	–	–
Non-Hispanic Black	202	–	–	–	–
Hispanic or Latino	139	–	–	–	–
Other/Unknown	257	–	–	–	–
2L CDKi					
Non-Hispanic White	1594	1.00 (ref)	–	1.00 (ref)	–
Non-Hispanic Black	221	0.67 (0.54–0.82)	< 0.001	0.80 (0.63–1.02)	0.069
Hispanic or Latino	180	0.79 (0.63–1.00)	0.051	0.90 (0.68–1.19)	0.446
Other/Unknown	413	0.98 (0.82–1.17)	0.838	1.08 (0.88–1.32)	0.465
2L Endocrine alone					
Non-Hispanic White	1056	1.00 (ref)	–	1.00 (ref)	–
Non-Hispanic Black	159	0.73 (0.58–0.91)	0.005	0.90 (0.69–1.17)	0.423
Hispanic or Latino	127	0.84 (0.65–1.09)	0.190	0.97 (0.72–1.32)	0.870
Other/Unknown	249	0.89 (0.74–1.09)	0.258	0.94 (0.75–1.17)	0.566
2L Everolimus					
Non-Hispanic White	168	1.00 (ref)	–	1.00 (ref)	–
Non-Hispanic Black	29	0.83 (0.55–1.27)	0.394	1.07 (0.68–1.70)	0.763
Hispanic or Latino	21	0.88 (0.54–1.43)	0.594	1.26 (0.73–2.16)	0.409
Other/Unknown	60	1.35 (0.98–1.87)	0.068	1.28 (0.89–1.84)	0.178

CDKi cyclin-dependent kinase 4/6 inhibitor; *cOR* crude odds ratio; *aOR* adjusted odds ratio

^a^Adjusted for age at metastatic diagnosis, stage at initial diagnosis, practice type, 1L treatment group, duration of 1L treatment, progression within 6 months of 1L start, ECOG status at 2L start, number and sites of metastasis and insurance group; Race reference group: Non-Hispanic White; 2L treatment reference group: 2L chemotherapy and others

### Real-world Overall Survival from 2L

**Fig. 2:**
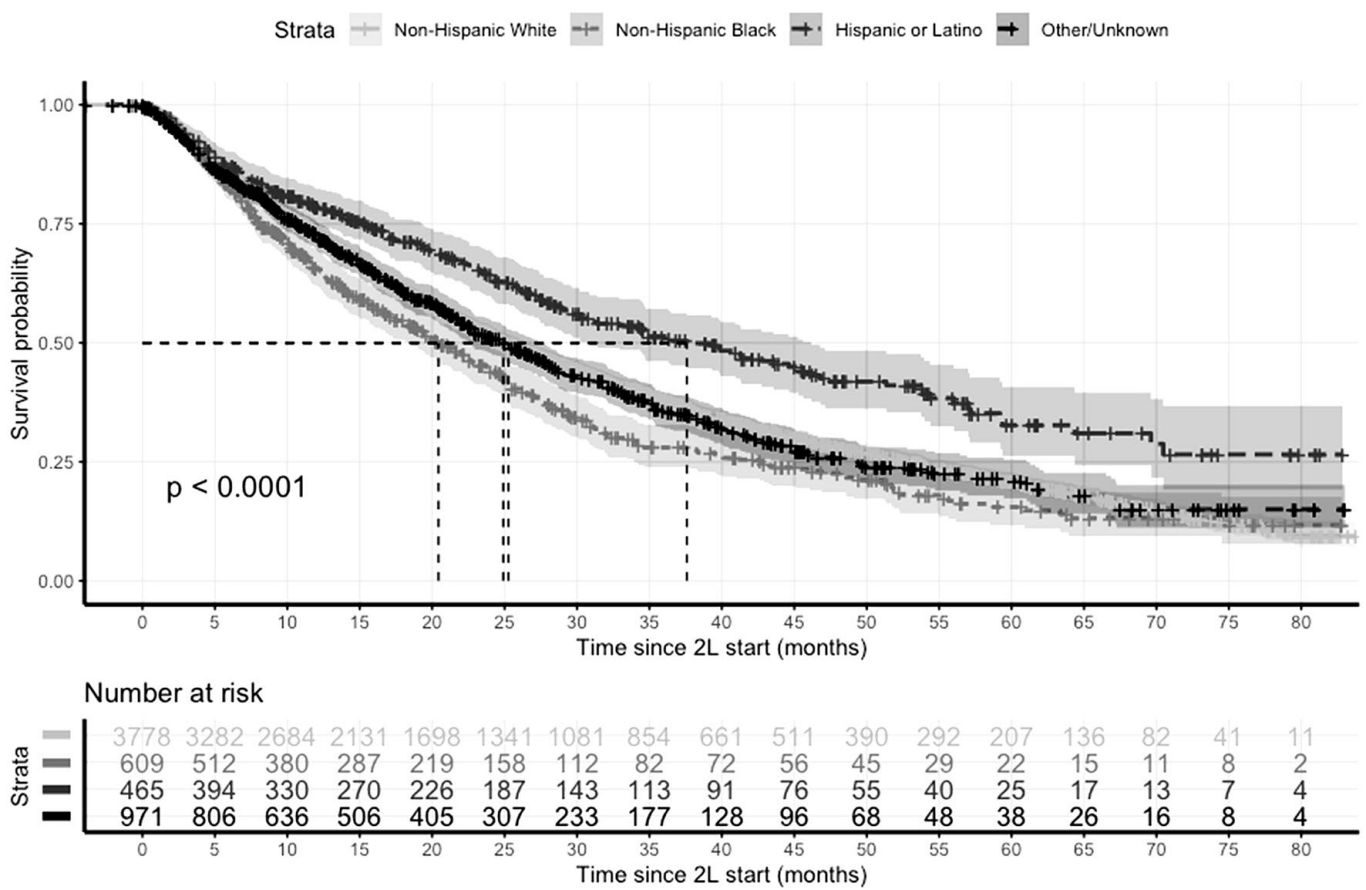
Kaplan Meier curve of rwOS since 2L start by race

Compared to NHW patients who had a median rwOS of 25.3 months (95% CI: 24.2–26.6), median rwOS was 20.4 months (95% CI: 18.3–23.7) among NHB patients, and 37.6 months (95% CI: 30.9–46.2) among Hispanic/Latino patients (Table 3, Fig. 2). Poorer rwOS among NHB patients was observed across all 2L treatment groups, except among those who received 2L chemotherapy or other therapies (Table 3). After adjusting for 2L treatment groups and other factors, the association between NHB and rwOS remained statistically significant (aHR: 1.16; 95% CI: 1.04–1.31). When stratified by 2L treatment groups, there is evidence that, compared to NHW patients receiving the same treatment, NHB patients who received endocrine monotherapy and CDKi both had 31% higher hazard of death (*p* = 0.010). In contrast, Hispanic/Latino patients had better rwOS than NHW patients overall (aHR = 0.70; 95% CI: 0.60—0.81), and among those who received endocrine monotherapy (aHR = 0.64; 95% CI: 0.48—0.85) and CDKi (aHR = 0.64; 95% CI: 0.48—0.85). Among patients who received 2L chemotherapy or other therapies, there is no difference in rwOS across any race/ethnicity groups. In sensitivity analysis, omitting ECOG status and insurance results in similar results, whereas only the association between rwOS and Hispanic/Latino patients (vs NHW) remains statistically significant when patients with missing ECOG or insurance are excluded (Supplemental Table 1).

**Table 3:** Associations between rwOS and race by 2L treatment group

	*N*	Median rwOS (95% CI)	Adjusted HR^a^ (95% CI)	*p*-value	*p*-int^c^
Overall					0.110
Non-Hispanic White	3792	25.3 (24.2–26.6)	1.00 (ref)	–	
Non-Hispanic Black	611	20.4 (18.3–23.7)	1.16^b^ (1.04–1.31)	0.009	
Hispanic or Latino	467	37.6 (30.9–46.2)	0.70^b^ (0.60–0.81)	< 0.001	
Other or unknown	979	24.9 (22.6–27.7)	1.03^b^ (0.93–1.13)	0.600	
Stratified					
2L Chemotherapy or other					
Non-Hispanic White	974	15.3 (14.3–17.2)	1.00 (ref)	–	
Non-Hispanic Black	202	15.6 (12.3–20.4)	0.97 (0.79–1.18)	0.727	
Hispanic or Latino	139	20.1 (16.0–27.7)	0.84 (0.65–1.09)	0.187	
Other or Unknown	257	15.0 (12.4–18.0)	1.07 (0.90–1.27)	0.449	
2L CDKi					
Non-Hispanic White	1594	34.5 (31.3–36.9)	1.00 (ref)	–	
Non-Hispanic Black	221	25.1 (22.4–30.9)	1.31 (1.07–1.61)	0.010	0.080
Hispanic or Latino	180	51.4 (37.6–NA)	0.64 (0.48–0.85)	0.002	0.246
Other or Unknown	413	35.8 (32.2–40.2)	0.92 (0.77–1.09)	0.338	0.220
2L Endocrine alone					
Non-Hispanic White	1056	25.0 (22.5–27.3)	1.00 (ref)	–	
Non-Hispanic Black	159	20.4 (15.9–26.8)	1.31 (1.07–1.62)	0.010	0.035
Hispanic or Latino	127	45.6 (30.2–59.4)	0.64 (0.48–0.85)	0.002	0.185
Other or Unknown	249	22.7 (19.5–26.4)	1.14 (0.95–1.36)	0.158	0.606
2L Everolimus combination					
Non-Hispanic White	168	21.8 (17.4–28.5)	1.00 (ref)	–	
Non-Hispanic Black	29	13.8 (5.7–31.2)	1.55 (0.91–2.65)	0.140	0.080
Hispanic or Latino	21	44.6 (21.4–NA)	0.60 (0.27–1.37)	0.230	0.541
Other or Unknown	60	20.0 (12.0–29.9)	1.17 (0.78–1.77)	0.451	0.719

^a^Adjusted for age at metastatic diagnosis, stage at initial diagnosis, practice type, insurance type, 1L treatment group, duration of 1L treatment, progression within 6 months of 1L start, ECOG performance status at 2L start,and number and sites of metastasis; Race reference group: Non-Hispanic White

^b^Overall survival analysis for all patients who received 2L treatment was additionally adjusted for 2L treatment group

^c^P-values for interaction term between race and 2L treatment groups were calculated in multivariate analysis

In exploratory analysis of stratifying patients by both 1L CDKi use and 2L treatment groups, NHB patients had worse rwOS compared to NHW patients, both among those who received 1L CDKi (aHR = 1.20; 95% CI: 0.97–1.49) and those who did not (aHR = 1.17; 95% CI: 1.03–1.34). Among the patients who received CDKi at 1L, no statistically significant difference in rwOS between race/ethnicity groups was observed across all 2L treatment groups. In contrast, among patients who did not receive 1L CDKi, NHB patients had 37% higher hazard of death if they received 2L CDKi (aHR = 1.37; 95% CI: 1.08–1.74), and 44% higher hazard of death if received 2L endocrine monotherapy (aHR = 1.44; 95% CI: 1.13–1.82; Table 4; Supplemental Fig. 2).

**Table 4:** Associations between rwOS and race stratified by 1L and 2L treatment

CDK 4/6 inhibitors at 1L	2L treatment	Non-Hispanic White		Non-Hispanic Black			Hispanic or Latino			Other/Unknown		
		*N*	HR	*N*	Adjusted HR^a^ (95% CI)	p-value	N	Adjusted HR^a^ (95% CI)	*p*-value	*N*	adjusted HR^a^ (95% CI)	*p*-value
Yes	Overall	1221	1.00 (ref)	181	1.20 (0.97–1.49)	0.091	131	0.94 (0.70–1.26)	0.672	343	1.00 (0.84–1.19)	0.995
	Stratified											
	Chemotherapy or others	360	1.00 (ref)	50	1.05 (0.73–1.51)	0.797	34	1.57 (0.99–2.49)	0.054	97	0.99 (0.74–1.31)	0.917
	CDKi	486	1.00 (ref)	73	1.18 (0.78–1.79)	0.423	64	0.77 (0.48–1.24)	0.285	148	0.75 (0.54–1.04)	0.080
	Endocrine alone	221	1.00 (ref)	34	1.03 (0.64–1.67)	0.901	15	NA	NA	54	1.39 (0.92–2.09)	0.119
	Everolimus combination^c^	124	1.00 (ref)	20	NA	NA	14	NA	NA	36	1.15 (0.67–1.95)	0.618
No	Overall	2601	1.00 (ref)	434	1.17 (1.03–1.34)	0.019	340	0.65 (0.54–0.77)	< 0.001	644	1.04 (0.92–1.17)	0.509
	Stratified											
	Chemotherapy or others	614	1.00 (ref)	152	0.94 (0.74–1.20)	0.643	105	0.71 (0.52–0.97)	0.030	160	1.15 (0.92–1.44)	0.228
	CDKi	1108	1.00 (ref)	148	1.37 (1.08–1.74)	0.010	116	0.56 (0.39–0.80)	0.002	265	0.95 (0.78–1.17)	0.645
	Endocrine alone	835	1.00 (ref)	125	1.44 (1.13–1.82)	0.003	112	0.66 (0.49–0.90)	0.008	195	1.08 (0.88–1.33)	0.438
	Everolimus combination^c^	44	1.00 (ref)	9	NA	NA	7	NA	NA	24	NA	NA

*CDKi* cyclin-dependent kinase 4/6 inhibitor; *CI* confidence interval; *HR* hazard ratio

^a^Adjusted for age at metastatic diagnosis, stage at initial diagnosis, practice type, insurance type, 1L treatment group, duration of 1L treatment, progression within 6 months of 1L start, ECOG performance status at 2L start,and number and sites of metastasis; Race reference group: Non-Hispanic White

^b^Overall survival analysis for all patients who received 2L treatment was additionally adjusted for 2L treatment group

^c^Multivariate analysis was not performed among the patients with 2L everolimus combination therapy due to small sample size

## Discussion

In this study, we did not observe statistically significant differences in the use of 2L CDKi across race/ethnicity groups among patients with HR+HER2- mBC, after adjusting for demographic and clinical factors. However, we found that NHB patients had poorer rwOS after 2L initiation, and the observed inequities in rwOS were mainly among the subset of women who did not receive CDKi at 1L. Our findings suggest that racial inequities in rwOS measured from the start of 2L treatment may be attributable to outcomes among women who do not receive CDKi during 1L treatment.

Racial differences in 2L treatment choices could be related to socioeconomic factors and access to care, potential differences in tumor biology (e.g. less targetable mutations, such as the PIK3CA mutation, for subsequent endocrine treatments, or possible provider concerns related to drug toxicities or efficacy. Pooled analysis from eight clinical trials among women with HR+HER2- advanced breast cancer reported that CDKi combination therapy is associated with higher risks of all-grade and grade 3 and 4 (G3-4) toxicities, including neutropenia, leukopenia, anemia and non-hematologic events [14]. However, whether NHB or Hispanic/Latino patients experience more toxicities than NHW patients when treated with CDKi is unknown as the clinical trials that demonstrated the efficacy of the CDKi did not enroll adequate numbers of NHB or Hispanic/Latino patients and rates of toxicities were not examined in the different racial and ethnic groups. Thus, without clinical trial data related to rates of toxicities in different racial and ethnic groups, it is possible that some providers may have increased concerns about toxicities and tolerance of CDKi in NHB or Hispanic/Latino patients based off their clinical experiences with these drugs, which could contribute to lower rates of 2L CDKi use in NHB or Hispanic/Latino compared to NHW patients. Additionally, it is possible that the increased monitoring for toxicities that comes with the use of CDKi could be a barrier to their use experience more among non-NHW patients, since they may face more adverse socioeconomic factors, such as insufficient or more stringent insurance coverage resulting in higher co-pays for visits and treatments, or challenges associated with the costs of o the medication itself, and transportation to follow-up appointments, clinic visits or lab tests. We found that NHB or Hispanic/Latino patients were more likely to have Medicaid or uninsured, suggesting that financial constraints may play a role in their ability to access treatment with CDKi and receive the supportive care needed to remain on treatment. While commercially insured patients may qualify for savings on the treatment, patients on Medicaid insurance can only obtain conditional support for palbociclib.

We found that the racial difference in rwOS measured after 2L initiation was driven by the subset of patients who did not receive CDKi at 1L. Meta-analysis from randomized clinical trials suggests that combination of CDKi and ET improves overall survival among patients with metastatic breast cancer in both 1L and 2L subgroups, compared to ET alone [13]. In this study, we found that about 40% of the patients treated in routine clinical practice in our study received CDKi as part of both 1L and 2L (Supplemental Fig. 1), although there is limited evidence on the efficacy of sequential CDKi treatment after progression and is the subject of ongoing or recently completed clinical trials (MAINTAIN study, TRINITI-1 study, PACE study, etc.). Among patients who received CDKi as part of 1L and 2L treatment, the estimated median rwOS was similar between race and ethnic groups in this context. On the other hand, among patients who received CDKi only at 2L but not at 1L, we found that NHB patients had worse rwOS after 2L CDKi initiation compared to White patients. This novel finding suggests that CDKi therapy may have a potentially larger impact as 1L among NHB patients, and administering CDKi earlier may help to reduce the racial inequities in survival outcomes among patients with HR+HER2− mBC. In addition, we observed better rwOS among Hispanic/Latino patients, which is consistent with previous population-based studies [21, 22], and known as “Hispanic Paradox” [23]. This observation has not been fully understood, but researchers have hypothesized that it could be due to selective immigration of healthy Hispanic/Latinos [24], the return of foregin-born Hispanic/Latinos to their native countries after diagnosis [25], and/or environmental and behavioral factors [25–27].

To our knowledge, this is the first study comparing treatment choices beyond front-line and corresponding survival outcomes across race/ethnicity groups among patients in the 2L setting for HR+HER2− mBC. Previous subgroups analysis by race among trial patients only reported Asian and non-Asian groups [14]. However, the difference in mortality is largest between NHB and NHW patients, and thus it is critically important that continued efforts are made to understand the drivers of this inequity and also implement solutions to mitigate it. Additionally, our study provides insight into treatment patterns in a patient cohort largely originated in community clinics, rather than large academic medical centers where the majority of clinical trial patients were recruited from. Our study therefore supplements current knowledge of real-world treatment patterns and outcomes among patients with HR+HER2− mBC by leveraging high quality contemporary electronic health record data, and we were able to assess the longitudinal treatment patterns throughout the patients treatment journey. In addition, we were able to adjust for critical clinical characteristics, including detailed drug categories, oral treatment information and progression events, that were curated with abstraction from unstructured documents. We had sufficient long-term follow-up from 2L treatment initiation, and we were also able to assess survival outcomes using high quality real-world composite mortality data in our cohort.

As a real-world retrospective cohort study, there are also some limitations. The treatment choices were not random. Although we adjusted for potential confounders including demographics, tumor characteristics and clinical factors at 1L, there is potential bias from confounding by unmeasured covariates. We are limited to information documented during the course of routine care. Our sensitivity analysis suggested that variables such as ECOG status and insurance might have been missing not at random, and the impact on missing values and observed racial differences warrant future investigations. Moreover, factors such as social network support and marital status that may also play a role in the patients’ treatment decisions and disease outcomes are not available. Further studies are needed to understand the contribution of those factors to inequities. In addition, although we only included patients who had a documented visit within 90 days of metastatic diagnosis, there is still a possibility that patients may have got treatment outside our network. Lastly, we were not able to include other race/ethnicity groups (e.g. Asian) in our analysis due to the small sample size in the patient population.

Our study is the first study to focus on the treatment choices and corresponding survival outcomes beyond the front-line among patients with HR+HER2− mBC. We found that the differences in treatment choices may be partially explained by tumor characteristics and socioeconomic status between race groups, and that administering CDKi earlier in the patient treatment journey may be associated with racial inequities in breast cancer survival outcomes. Our results supplement existing evidence and highlight a need for further insights in this patient population.

## Supplementary Information

Below is the link to the electronic supplementary material.

Supplementary file1 (DOCX 322 KB)

## Data Availability

The data that support the findings of this study have been originated by Flatiron Health, Inc. These de-identified data may be made available upon request, and are subject to a license agreement with Flatiron Health; interested researchers should contact <www.DataAccess@flatiron.com> to determine licensing terms.
