# Supplementary material for: Racial inequities in second-line treatment and overall survival among patients with metastatic breast cancer
Source: Breast Cancer Res Treat. 2022 Aug 26;196(1):163–73. doi: 10.1007/s10549-022-06701-5 (PMC9550747; doi:10.1007/s10549-022-06701-5)
Supplement: Supplementary file 1 — Supplementary file1 (DOCX 322 KB) [file 10549_2022_6701_MOESM1_ESM.docx]

**Racial disparities in second-line treatment and overall survival among patients with metastatic breast cancer**

Kristen D. Whitaker, Xiaoliang Wang, Mustafa Ascha, Timothy N. Showalter, Heather G. Lewin, Gregory S. Calip, Lori J. Goldstein

## **SUPPLEMENT**

## Supplemental Figure 1. Proportion of 2L treatment by race/ethnicity group, stratified by 1L CDK 4/6 inhibitors use


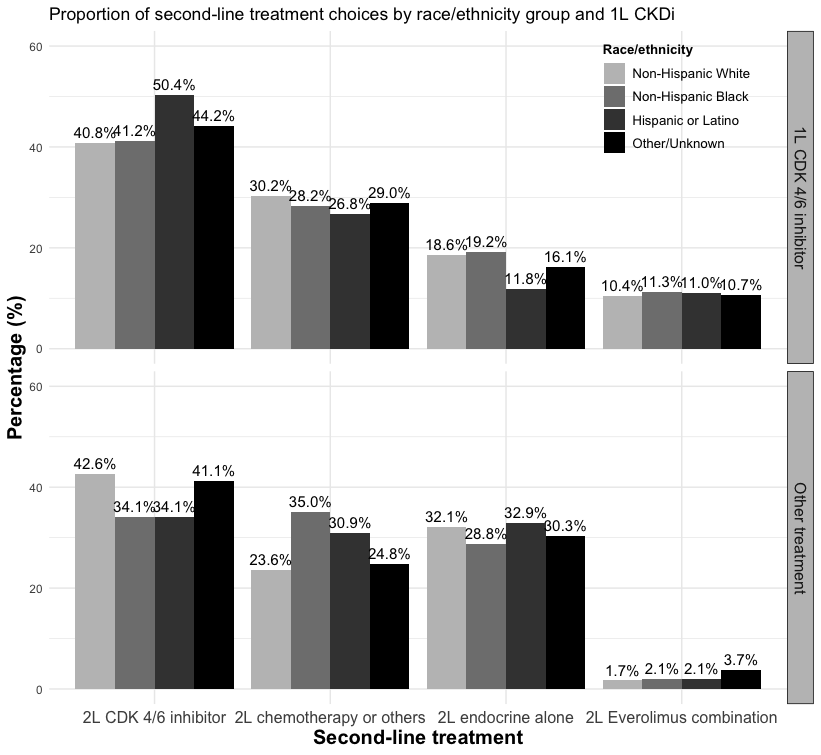


## Supplemental Figure 2. Kaplan-Meier curve of real-world overall survival by race and 1L CDK 4/6 inhibitors use

### 1L CDK 4/6 use


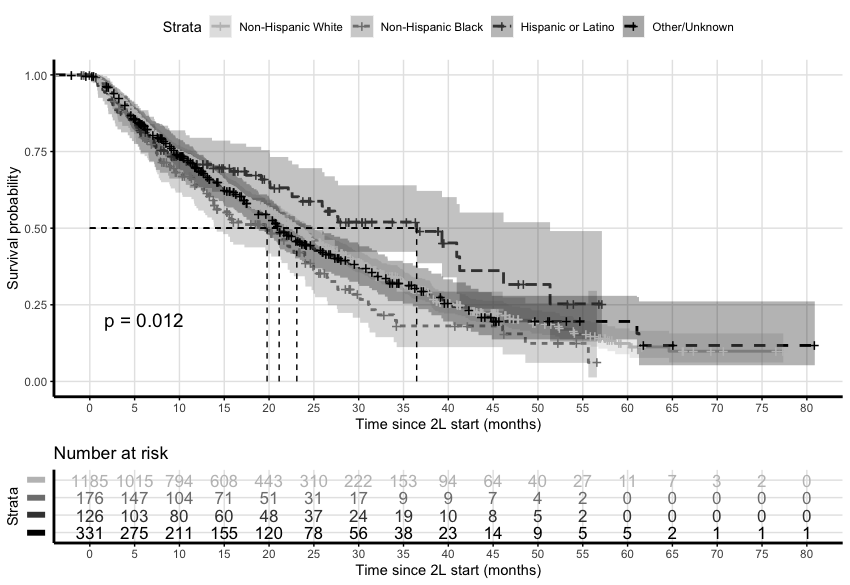


### 1L others


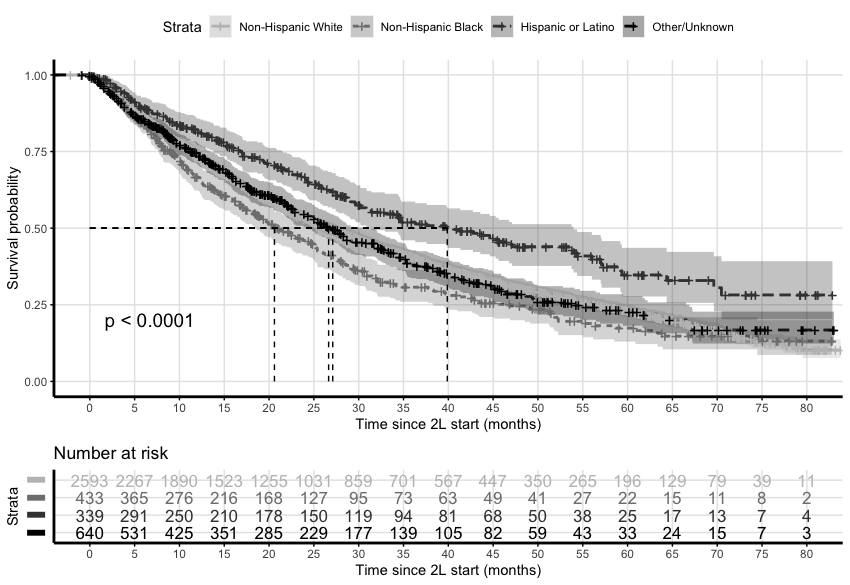


##

##

## Supplemental Table 1. Sensitivity analyses of associations between race and likelihood of receiving different 2L treatment

| **Race** | **Primary analysis^a^** | | |  | **Omit variables^b^** | |  | **Complete case analysis^a,c^** | | |
| --- | --- | --- | --- | --- | --- | --- | --- | --- | --- | --- |
|  | **N** | **OR (95% CI)** | **p-value** |  | **OR (95% CI)** | **p-value** |  | **N** | **OR (95% CI)** | **p-value** |
| **2L chemotherapy or other (reference group)** | | | | | | | | | | |
| Non-Hispanic White | 974 | - | - |  | - | - |  | 636 | - | - |
| Non-Hispanic Black | 202 | - | - |  | - | - |  | 132 | - | - |
| Hispanic or Latino | 139 | - | - |  | - | - |  | 70 | - | - |
| Other/Unknown | 257 | - | - |  | - | - |  | 151 | - | - |
| **2L CDKi** |  |  |  |  |  |  |  |  |  |  |
| Non-Hispanic White | 1594 | 1.00 (ref) | - |  | 1.00 (ref) | - |  | 1046 | 1.00 (ref) | - |
| Non-Hispanic Black | 221 | 0.80 (0.63 - 1.02) | 0.069 |  | 0.82 (0.65 - 1.03) | 0.090 |  | 151 | 0.86 (0.65 - 1.13) | 0.279 |
| Hispanic or Latino | 180 | 0.90 (0.68 - 1.19) | 0.446 |  | 0.91 (0.70 - 1.17) | 0.452 |  | 94 | 0.93 (0.66 - 1.33) | 0.708 |
| Other/Unknown | 413 | 1.08 (0.88 - 1.32) | 0.465 |  | 1.06 (0.87 - 1.28) | 0.579 |  | 241 | 1.05 (0.82 - 1.35) | 0.683 |
| **2L Endocrine alone** |  |  |  |  |  |  |  |  |  |  |
| Non-Hispanic White | 1056 | 1.00 (ref) | - |  | 1.00 (ref) | - |  | 658 | 1.00 (ref) | - |
| Non-Hispanic Black | 159 | 0.90 (0.69 - 1.17) | 0.423 |  | 0.93 (0.72 - 1.19) | 0.565 |  | 96 | 0.91 (0.67 - 1.24) | 0.541 |
| Hispanic or Latino | 127 | 0.97 (0.72 - 1.32) | 0.870 |  | 1.00 (0.76 - 1.33) | 0.983 |  | 51 | 0.85 (0.57 - 1.27) | 0.430 |
| Other/Unknown | 249 | 0.94 (0.75 - 1.17) | 0.566 |  | 0.98 (0.79 - 1.22) | 0.874 |  | 133 | 0.98 (0.74 - 1.29) | 0.879 |
| **2L Everolimus** |  |  |  |  |  |  |  |  |  |  |
| Non-Hispanic White | 168 | 1.00 (ref) | - |  | 1.00 (ref) | - |  | 113 | 1.00 (ref) | - |
| Non-Hispanic Black | 29 | 1.07 (0.68 - 1.70) | 0.763 |  | 1.20 (0.77 - 1.85) | 0.426 |  | 20 | 1.18 (0.70 - 1.99) | 0.532 |
| Hispanic or Latino | 21 | 1.26 (0.73 - 2.16) | 0.409 |  | 1.26 (0.76 - 2.08) | 0.380 |  | 12 | 1.26 (0.66 - 2.41) | 0.485 |
| Other/Unknown | 60 | 1.28 (0.89 - 1.84) | 0.178 |  | 1.47 (1.05 - 2.07) | 0.027 |  | 30 | 1.24 (0.80 - 1.95) | 0.338 |
| CDKi=cyclin-dependent kinase 4/6 inhibitor; cOR=crude odds ratio; aOR=adjusted odds ratio | | | | | | | |  |  |  |

^a^ Adjusted for age at metastatic diagnosis, stage at initial diagnosis, practice type, 1L treatment group, duration of 1L treatment, progression within 6 month of 1L start, ECOG status at 2L start, number and sites of metastasis and insurance group; Race reference group: White; 2L treatment reference group: 2L chemotherapy and others.

^b^ Adjusted for age at metastatic diagnosis, stage at initial diagnosis, practice type, 1L treatment group, duration of 1L treatment, progression within 6 month of 1L start, and number and sites of metastasis; Race reference group: White; 2L treatment reference group: 2L chemotherapy and others.

^c^ Patients with missing ECOG status or insurance group are excluded from the analysis.

## Supplemental Table 2 Sensitivity analysis for the associations between rwOS and race by 2L treatment group

|  | **Primary analysis^a^** | | |  | | **Omit variables^b^** | |  | **Complete case analysis^a,c^** | | | |
| --- | --- | --- | --- | --- | --- | --- | --- | --- | --- | --- | --- | --- |
|  | **N** | **adjusted HR**  **(95% CI)** | **p-value** |  | | **adjusted HR**  **(95% CI)** | **p-value** |  | **N** | | **adjusted HR**  **(95% CI)** | **p-value** |
| **Overall** |  |  |  |  |  | |  |  | |  |  |  |
| Non-Hispanic White | 3792 | 1.00 (ref) | - |  | 1.00 (ref) | | - |  | | 2453 | 1.00 (ref) | - |
| Non-Hispanic Black | 611 | 1.16^d^ (1.04 - 1.31) | 0.009 |  | 1.18^d^ (1.06 - 1.32) | | 0.003 |  | | 399 | 1.05^d^ (0.91 - 1.21) | 0.500 |
| Hispanic or Latino | 467 | 0.70^d^ (0.60 - 0.81) | <0.001 |  | 0.71^d^ (0.61 - 0.82) | | <0.001 |  | | 227 | 0.75^d^ (0.61 - 0.92) | 0.006 |
| Other or Unknown | 979 | 1.03^d^ (0.93 - 1.13) | 0.600 |  | 1.04^d^ (0.94 - 1.14) | | 0.400 |  | | 555 | 1.03^d^ (0.91 - 1.17) | 0.600 |
| **Stratified** |  |  |  |  |  | |  |  | |  |  |  |
| 2L Chemotherapy or other |  |  |  |  |  | |  |  | |  |  |  |
| Non-Hispanic White | 974 | 1.00 (ref) | - |  | 1.00 (ref) | | - |  | | 636 | 1.00 (ref) | - |
| Non-Hispanic Black | 202 | 0.97 (0.79 - 1.18) | 0.727 |  | 0.97 (0.80 - 1.18) | | 0.775 |  | | 132 | 0.84 (0.65 - 1.08) | 0.172 |
| Hispanic or Latino | 139 | 0.84 (0.65 - 1.09) | 0.187 |  | 0.88 (0.70 - 1.11) | | 0.284 |  | | 70 | 0.85 (0.61, 1.19) | 0.343 |
| Other or Unknown | 257 | 1.07 (0.90 - 1.27) | 0.449 |  | 1.06 (0.90 - 1.26) | | 0.484 |  | | 151 | 0.92 (0.73 - 1.15) | 0.471 |
| 2L CDKi |  |  |  |  |  | |  |  | |  |  |  |
| Non-Hispanic White | 1594 | 1.00 (ref) | - |  | 1.00 (ref) | | - |  | | 1046 | 1.00 (ref) | - |
| Non-Hispanic Black | 221 | 1.31 (1.07 - 1.61) | 0.010 |  | 1.33 (1.09 - 1.62) | | 0.005 |  | | 151 | 1.18 (0.90 - 1.54) | 0.220 |
| Hispanic or Latino | 180 | 0.64 (0.48 - 0.85) | 0.002 |  | 0.64 (0.49 - 0.84) | | 0.001 |  | | 94 | 0.64 (0.44 - 0.93) | 0.019 |
| Other or Unknown | 413 | 0.92 (0.77 - 1.09) | 0.338 |  | 0.94 (0.80 - 1.11) | | 0.482 |  | | 241 | 0.97 (0.77 - 1.21) | .765 |
| 2L Endocrine alone |  |  |  |  |  | |  |  | |  |  |  |
| Non-Hispanic White | 1056 | 1.00 (ref) | - |  | 1.00 (ref) | | - |  | | 658 | 1.00 (ref) | - |
| Non-Hispanic Black | 159 | 1.31 (1.07 - 1.62) | 0.010 |  | 1.33 (1.08 - 1.63) | | 0.006 |  | | 96 | 1.26 (0.95 - 1.67) | 0.107 |
| Hispanic or Latino | 127 | 0.64 (0.48 - 0.85) | 0.002 |  | 0.63 (0.48 - 0.82) | | <0.001 |  | | 51 | 0.71 (0.45 - 1.13) | 0.148 |
| Other or Unknown | 249 | 1.14 (0.95 - 1.36) | 0.158 |  | 1.13 (0.95 - 1.35) | | 0.167 |  | | 133 | 1.19 (0.93 - 1.52) | 0.157 |
| 2L Everolimus combination |  |  |  |  |  | |  |  | |  |  |  |
| Non-Hispanic White | 168 | 1.00 (ref) | - |  | 1.00 (ref) | | - |  | | 113 | 1.00 (ref) | - |
| Non-Hispanic Black | 29 | 1.55 (0.91 - 2.65) | 0.140 |  | 1.40 (0.85 - 2.30) | | 0.185 |  | | 20 | 1.71 (0.83 - 3.52) | 0.148 |
| Hispanic or Latino | 21 | 0.60 (0.27 - 1.37) | 0.230 |  | 0.58 (0.26 - 1.30) | | 0.183 |  | | 12 | 0.32 (0.09 - 1.09) | 0.068 |
| Other or Unknown | 60 | 1.17 (0.78 - 1.77) | 0.451 |  | 1.28 (0.88 - 1.88) | | 0.195 |  | | 30 | 0.96 (0.56 - 1.64) | 0.879 |

^a^ Adjusted for age at metastatic diagnosis, stage at initial diagnosis, practice type, insurance type, 1L treatment group, duration of 1L treatment, progression within 6 month of 1L start, ECOG performance status at 2L start ,and number and sites of metastasis; Race reference group: White.

^b^ Adjusted for age at metastatic diagnosis, stage at initial diagnosis, practice type, 1L treatment group, duration of 1L treatment, progression within 6 month of 1L start, and number and sites of metastasis; Race reference group: White; 2L treatment reference group: 2L chemotherapy and others.

^c^ Patients with missing ECOG status or insurance group are excluded from the analysis.

^d^ Overall survival analysis for all patients who received 2L treatment was additionally adjusted for 2L treatment group.
